# “It's just not easy to understand”: A mixed methods study of health insurance literacy and insurance plan decision‐making in cancer survivors

**DOI:** 10.1002/cam4.6133

**Published:** 2023-05-23

**Authors:** Courtney P. Williams, Heather N. Platter, Amy J. Davidoff, Robin C. Vanderpool, Maria Pisu, Janet S. de Moor

**Affiliations:** ^1^ Divison of Preventive Medicine University of Alabama at Birmingham Birmingham Alabama USA; ^2^ Division of Cancer Control and Population Sciences National Cancer Institute Rockville Maryland USA

**Keywords:** cancer survivorship, decision‐making, health insurance literacy, mixed methods, out‐of‐pocket cost

## Abstract

**Background:**

Understanding cancer survivors' health insurance decision‐making is needed to improve insurance choice, potentially resulting in reduced financial hardship.

**Methods:**

This explanatory mixed methods study assessed health insurance decision‐making in cancer survivors. Health Insurance Literacy Measure (HILM) captured HIL. Quantitative eye‐tracking data collected from two simulated health insurance plan choice sets gauged dwell time (seconds), or interest, in benefits. Dwell time differences by HIL were estimated using adjusted linear models. Qualitative interviews explored survivors' insurance decision‐making choices.

**Results:**

Cancer survivors (*N* = 80; 38% breast cancer) had a median age of 43 at diagnosis (IQR 34–52). When comparing traditional and high‐deductible health plans, survivors were most interested in drug costs (median dwell time 58 s, IQR 34–109). When comparing health maintenance organization and preferred provider organization plans, survivors were most interested in test/imaging costs (40s, IQR 14–67). Survivors with low versus high HIL had more interest in deductible (*β* = 19 s, 95% CI 2–38) and hospitalization costs (*β* = 14 s, 95% CI 1–27) in adjusted models. Survivors with low versus high HIL more often ranked out‐of‐pocket (OOP) maximums and coinsurance as the most important and confusing benefits, respectively. Interviews (*n* = 20) revealed survivors felt alone “to do their own research” about insurance choices. OOP maximums were cited as the deciding factor since it is “how much money is going to be taken out of my pocket.” Coinsurance was considered “rather than a benefit, it's a hindrance.”

**Conclusion:**

Interventions to aid in health insurance understanding and choice are needed to optimize plan choice and potentially reduce cancer‐related financial hardship.

## Graphical abstract

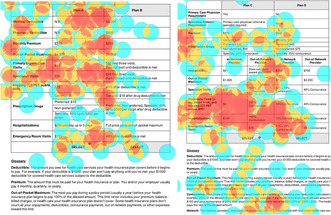

## 1 BACKGROUND

Financial hardship is a well‐documented, yet growing issue for individuals with cancer during active treatment and survivorship. High out‐of‐pocket (OOP) costs which frequently accompany cancer diagnosis and treatment are associated with psychological distress, material economic hardship, and adverse behavioral coping mechanisms such as delaying or foregoing recommended healthcare due to perceived cost.^1^, ^2^, ^3^ For cancer survivors, financial hardship may be exacerbated by low health insurance literacy.^4^, ^5^, ^6^ Due to complex variation in benefits,^7^ proficient health insurance literacy is important for choosing a health insurance plan well‐suited to an individual's healthcare needs. Health insurance literacy is defined as (1) the ability and confidence to understand health insurance terms and costs, (2) select the most appropriate individualized plan based on predicted utilization and cost, and (3) use the plan after enrollment.^8^ However, health insurance literacy is low among the general American public. In a nationally representative survey of 1292 U.S. adults assessing health insurance terms and concepts, only 4% of respondents answered all 10 questions correctly, and 8% gave no correct answers at all.^9^ Furthermore, half of the participants were unable to calculate potential OOP costs for a hospital stay and 84% were unable to do so for an out‐of‐network lab test.

For cancer survivors, low health insurance literacy may impact the ability to choose the best health insurance plan to facilitate access to preferred providers and recommended tests, procedures, and follow‐up care while minimizing high potential healthcare costs. This could put individuals at risk of financial hardship and its associated adverse outcomes.^10^ In our previous study evaluating health insurance literacy in women with metastatic breast cancer, half of surveyed patients had low health insurance literacy. Those patients more often skipped recommended medical care and had difficulty paying for food, heat, or rent when undergoing cancer treatment when compared to patients with high health insurance literacy. This suggests an association between health insurance literacy and the ability to pay for care and other necessary expenses.^11^ However, little is known about how cancer survivors make health insurance plan choices.

To address this knowledge gap, we used a novel eye‐tracking methodology coupled with qualitative interviews with the aim of better understanding health insurance plan benefits important to decision‐making in cancer survivors with differing health insurance literacy. Use of eye‐tracking methodologies allows for examination of cancer survivors' attention to specific components of health insurance plans and how attention may differ by health insurance literacy. This information is needed to inform interventions which improve insurance choice, potentially resulting in expanded healthcare access and reduced financial hardship.

## 2 METHODS

### 2.1 Study design and sample

This sequential, explanatory mixed methods study (QUANT➔QUAL)^12^ integrated eye‐tracking (collected December 2021) and interview data (collected March–April 2022) from a national sample of cancer survivors recruited from a market research company. Survivors were contacted via e‐mail and asked to complete a web‐based screener to determine eligibility. Eligible survivors were <5 years from their cancer diagnosis, privately or commercially insured when receiving cancer treatment, and between the ages of 26 and 60 at diagnosis (ineligible for dependent child coverage and Medicare). If eligible, survivors were contacted via phone to confirm screening criteria. Proportional quota sampling was then used to choose survivors for inclusion to ensure diversity based on health insurance literacy level, race and ethnicity, and cancer type. Survivors who did not have a webcam‐enabled computer or laptop, wore glasses, or had a history of seizures were excluded due to webcam eye‐tracking guidelines. All survivors gave verbal consent prior to study participation. Survivors were compensated $25 and $75 for completion of the eye‐tracking and interviews, respectively. This study was deemed as exempt human subjects research by the National Institutes of Health Office of Institutional Review Board Operations.

### 2.2 Health insurance literacy

Survivors' health insurance literacy was captured during study screening using the 21‐item Health Insurance Literacy Measure (HILM), which assesses confidence in and behaviors surrounding choosing and using health insurance.^13^ Scores range from 21 to 84, with higher scores indicating higher health insurance literacy. Survivors with HILM scores ≤60 were considered as having low health insurance literacy.^10^ Self‐reported survivor sociodemographics (age, race and ethnicity, education, sex, and household income) and cancer‐related characteristics (diagnosis age, cancer type, cancer treatment, health insurance coverage during cancer treatment) were also captured during screening.

### 2.3 Health insurance decision‐making

**FIGURE 1:**
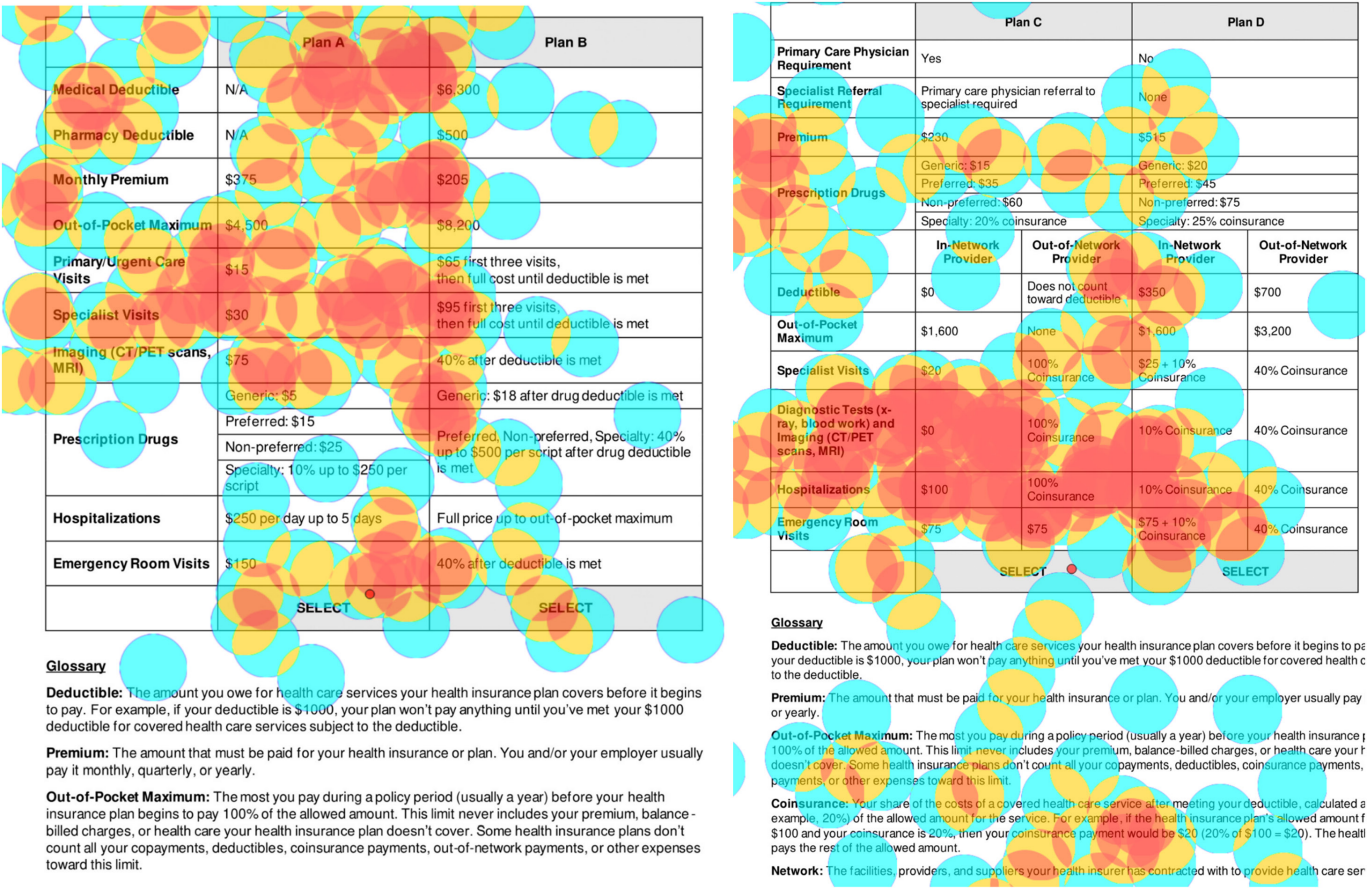
Example choice task heat maps, including a choice between a traditional (Plan A) and high‐deductible healthcare plan (Plan B), and between a health maintenance organization plan (Plan C) and preferred provider organization plan (Plan D). Circles represent respondent areas of interest measured by eye fixations, with the warmer colored circles indicating longer duration of fixation (more dwell time; red, orange, yellow), and the cooler colored circles indicating shorter durations of fixation (less dwell time; blue).

Survivors completed two health insurance plan decision tasks using the RealEye.io online webcam eye‐tracking platform, which captured eye movements during task completion. After successful webcam calibration for accurate data capture,^14^ survivors were asked to imagine they were about to start cancer treatment and had to switch health insurance plans. Survivors were then directed to view health insurance plans from two simulated insurance plan choice sets (Figure 1). Plans included benefits relevant to individuals diagnosed with cancer. One health insurance plan set included a choice between a traditional and high‐deductible health plan (HDHP) to simulate different cost sharing structures (Plans A and B; Figure 1). The second health insurance plan set included a choice between a health maintenance organization (HMO) and preferred provider organization (PPO) plan to simulate different roles of coverage in provider access (Plans C and D; Figure 1). A glossary of plan benefit definitions was provided under each plan choice set. Eye‐tracking data collected during the choice set tasks included dwell time, measured by the duration (in seconds) of all fixations (period of no eye movement exceeding a certain timeframe) on an area of interest (spatial region on screen).^15^ We defined areas of interest as individual plan benefits, including the costs of each plan deductible, premium, OOP maximum, primary/urgent care visits, specialist visits, imaging, diagnostic tests, prescription drugs, hospitalizations, emergency room visits, or gatekeeper items including primary care physician or specialist referral requirements. After completing the health insurance plan decision tasks, survivors were asked to choose the most important and confusing health insurance plan benefit when starting cancer treatment. These data were collected to differentiate dwell time as a product of importance or processing difficulty.

### 2.4 Explanatory interviews

Participants completing the health insurance decision tasks were purposively selected for qualitative interviews, representing a balance of cancer types, health insurance literacy status, and non‐missing eye‐tracking data. Selected participants engaged in 30‐min, semi‐structured interviews with experts in decision‐making (CW) and qualitative (HP) methodologies using a virtual video platform. An interview guide was created based on the eye‐tracking data to explore health insurance plan decision‐making. Interviews were recorded and transcribed for analysis.

### 2.5 Statistical analysis

Median (IQR) time (seconds) spent looking at insurance plan areas of interest in the two health insurance decision tasks was compared overall and between those with high and low health insurance literacy. Primary quantitative outcomes included (1) time (seconds) spent looking at insurance plan benefits, and the (2) most important and (3) most confusing insurance plan benefit during decision‐making when starting cancer treatment. Multivariable generalized linear models estimated differences in time spent looking at insurance plan benefits for those with high and low health insurance literacy using beta coefficients (*β*) and corresponding 95% confidence intervals (CI). Models were adjusted for diagnosis age, race and ethnicity, and sex. Proportions of survivors reporting insurance plan benefits as most important and confusing were compared overall and between those with high and low health insurance literacy.

To analyze interview data, two independent coders (CW and HP) developed a preliminary codebook based on the interview guide. Using deductive content analysis, keywords and themes were mapped to codes using focused coding. Intercoder reliability was confirmed after 20% of the transcripts were coded (*κ* = 0.9).

## 3 RESULTS

### 3.1 Sample characteristics

Of 23,783 web‐based study screeners e‐mailed to cancer survivors, 1150 were completed (5%), 771 survivors were rescreened via phone (67% of those who completed web‐based screeners), 282 were eligible for study inclusion (25%), 97 were consented (8%), and 80 survivors completed the eye‐tracking survey and were included in our analytic sample (7%; Figure S1). Survivors were a median 43 years old at diagnosis (IQR 34–52), 65% were female, 59% were non‐Hispanic White, 84% had earned a college degree or higher, and 69% had annual household incomes ≥$75,000 (Table 1). The largest proportion of survivors were previously diagnosed with breast (38%) or colorectal (13%) cancer and had a treatment history of surgery (75%) and/or systemic therapy (75%). The median health insurance literacy score in our sample was 60 (IQR 50–70). Survivors with lower health insurance literacy were more often Hispanic/Latino, had less than a college degree, or had annual household incomes <$75,000 (Table 1).

**TABLE 1:** Respondent demographics and clinical characteristics (*N* = 80).

	Total *N* = 80	High health insurance literacy (*n* = 40)	Low health insurance literacy (*n* = 40)
	*n* (%)	*n* (%)	*n* (%)
Age at diagnosis (median, IQR)	43 (34–52)	43 (33–53)	44 (36–51)
Sex			
Female	52 (65)	26 (50)	26 (50)
Male	28 (35)	14 (50)	14 (50)
Race and ethnicity			
Non‐Hispanic White	47 (59)	23 (49)	24 (51)
Non‐Hispanic Black	15 (19)	9 (60)	6 (40)
Hispanic/Latino	10 (13)	4 (40)	6 (60)
Other	8 (10)	4 (50)	4 (50)
Education level			
<College degree	13 (16)	5 (38)	8 (62)
≥College degree	67 (84)	35 (52)	32 (48)
Annual household income			
<$75,000	25 (31)	10 (40)	15 (60)
≥$75,000	55 (69)	30 (55)	25 (45)
Health insurance status			
Private or employer sponsored	64 (80)	29 (45)	35 (55)
ACA exchange or other	16 (20)	11 (69)	5 (31)
Cancer type			
Breast	30 (38)	15 (50)	15 (52)
Colorectal	10 (13)	8 (80)	2 (20)
Genitourinary	4 (5)	1 (25)	3 (75)
Gynecologic	7 (9)	5 (71)	2 (29)
Lung	7 (9)	4 (57)	3 (43)
Other	22 (28)	7 (32)	15 (68)
Cancer treatment received			
Surgery	60 (75)	29 (48)	31 (52)
Radiation therapy	44 (55)	24 (55)	20 (45)
Systemic therapy	60 (75)	34 (57)	26 (43)
Clinical trial	2 (3)	1 (50)	1 (50)
Health insurance literacy score (median, IQR)	61 (50–70)	70 (63–75)	50 (45–57)

Abbreviations: ACA, Affordable Care Act; IQR, interquartile range.

### 3.2 Insurance plan decision‐making

**FIGURE 2:**
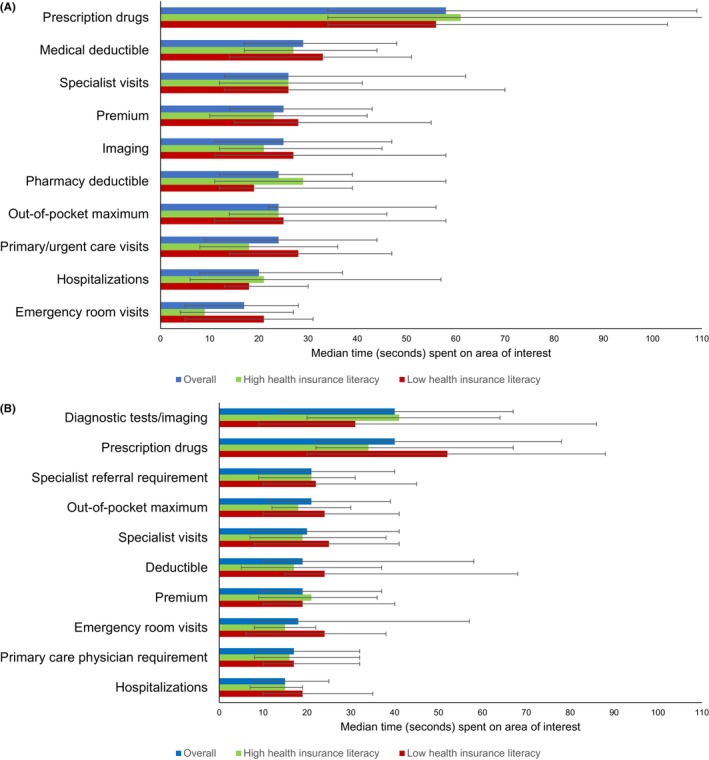
Median (interquartile range) time spent looking at plan benefit areas of interest overall and by health insurance literacy when comparing between (A) a traditional and high‐deductible plan, and (B) a health maintenance organization and preferred provider organization plan (*N* = 80).

In the health insurance decision task comparing a traditional plan and HDHP, survivors spent the most time comparing benefit costs related to prescription drugs (median dwell time 58 s, IQR 34–109 s), medical deductibles (29 s, IQR 17–48), and specialist visits (26 s, IQR 13–62; Figure 2A). When compared to those with high health insurance literacy, survivors with low health insurance literacy spent more time looking at the cost of emergency room visits (low: 21 s, IQR 5–31; high: 9 s, IQR 4–27) and primary or urgent care visits (low: 28 s, IQR 14–47; high: 18 s, IQR 8–36). In multivariable models, survivors with high and low health insurance literacy did not differ in the time spent looking at insurance plan benefits.

In the health insurance decision task comparing an HMO and PPO plan, survivors spent the most time comparing benefit costs related to prescription drugs (median dwell time 40 s, IQR 22–78), diagnostic tests and imaging (40 s, IQR 14–67), and specialist referral requirements (21 s, IQR 9–40; Figure 2B). Survivors with low health insurance literacy spent more time looking at the cost of prescription drugs (52 s, IQR 20–88) compared to those with high health insurance literacy (34 s, IQR 22–67). In multivariable models, survivors with low health insurance literacy spent more time looking at cost of deductibles (*β* = 19 s, 95% CI 2–38) and hospitalizations (*β* = 14 s, 95% CI 1–27) when compared to those with high health insurance literacy.

**FIGURE 3:**
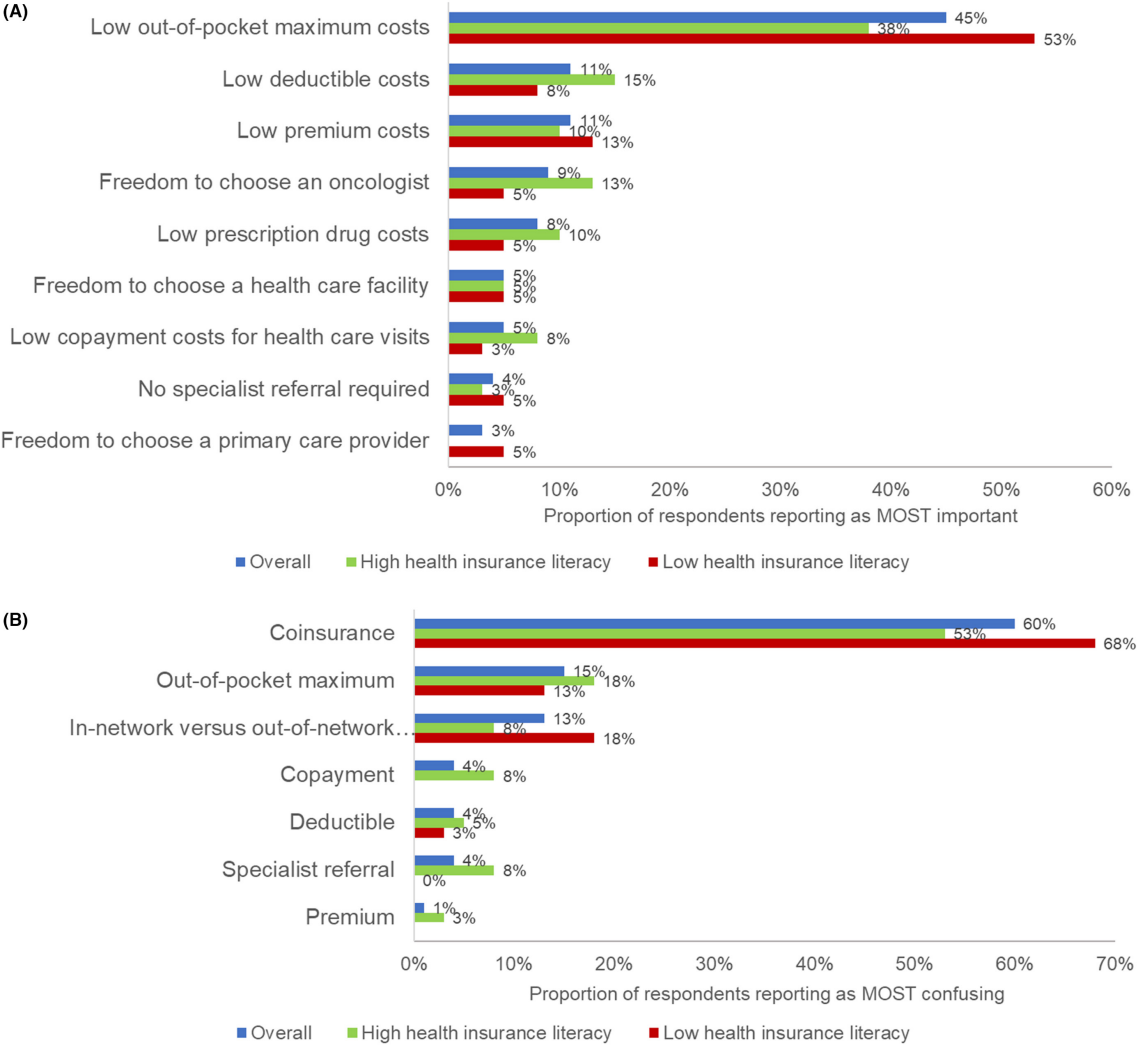
Respondent ranking of plan benefits (A) most important, and (B) most confusing when choosing a health insurance plan during cancer treatment (*N* = 80).

In ranking plan benefits important when making a health insurance plan choice, the most important plan benefit to survivors with both high and low health insurance literacy was low OOP maximum costs, though survivors with low health insurance literacy more often ranked this benefit as most important compared to those with high health insurance literacy (53% vs. 38%; Figure 3A). The most confusing plan benefit to survivors was coinsurance. Survivors with low health insurance literacy more often ranked this benefit as most confusing compared to those with high health insurance literacy (68% vs. 53%; Figure 3B). Survivors with low compared to high health insurance literacy also more often ranked differentiating between in‐network versus out‐of‐network providers as the most confusing plan benefit (18% vs. 8%).

### 3.3 Explanatory interviews

Cancer survivors selected for qualitative interviews (*n* = 20; *n* = 6 breast, 3 colorectal, 2 genitourinary, 2 gynecologic, 2 lung, 5 other cancer types) had a median age of 49 at diagnosis (IQR 37–53), 65% female, 75% non‐Hispanic White, 90% earned a college degree or higher, and had a median health insurance literacy score of 58 (IQR 49–63). Interviews revealed main themes related to (1) health insurance plan decision‐making and (2) issues post‐health insurance plan choice (Table 2).

**TABLE 2:** Qualitative interview themes, subthemes, codes, and prominent quotations.

Theme	Subtheme	Code	Quote
Health Insurance Plan Decision‐Making	Knowledge	Benefit Understanding	“In the end I would prefer to know the most that I'm going to have to pay.” (Regarding out‐of‐pocket maximums)
		Benefit Confusion	“Because honestly, I don't know if I am entirely sure what [coinsurance] means.” (Regarding coinsurance)
	Trade‐Offs	Out‐of‐Pocket Cost	“For me, it's all about the least amount of money out‐of‐pocket. Sometimes I'll select the most expensive plan if it means less money out of my pocket when I go [to the doctor] every time.” “I would rather pay a little more monthly because I know my wife and I are both getting up in years. We are going to see more doctors.”
		Specialist Referral Requirements	“Unless I have to go to a specialist, I try not to, to be honest with you.” “It's a waste of time. It's just a waste to get the [specialist] referral.”
Issues Post‐Health Insurance Plan Choice	Usability	Cost Confusion	“Honestly there was no discussion [about cost].”
		Lack of Assistance	“I was just kind of left to deal with my health insurance company myself.”

#### 3.3.1 Health insurance plan decision‐making: Knowledge

When asked to explain their decision‐making when choosing between health insurance plans, survivors used their baseline knowledge of benefits. For example, survivors heavily relied on understanding the OOP maximum when making insurance decisions, stating “In the long‐term, about how much money is going to be taken out of my pocket? So, because of that vast difference, that's why I selected the [traditional plan].” Survivors also emphasized the importance of understanding a plan's OOP maximum due to the high cost of treating their cancer, stating “My max out of pocket was cheaper…I have cancer. [I] would have to pay.”

Conversely, survivors considered the benefit design of coinsurance confusing, stating “I don't understand it at all. Oh, I have to use my deductible first, okay, then you'll kick in?… Rather than a benefit, it's a hindrance. There's no benefit I can see for having coinsurance.” Even survivors experienced with insurance did not fully understand coinsurance, stating “I'm in human resources and benefits just confuse the holy heck out of me. So, coinsurance, I hear that. And I go, ‘Stay away.’ I didn't even want to touch that with a 10‐foot pole.” Other survivors were confused about the concept of coinsurance itself, saying “That's kind of ambiguous, coinsurance. Does that mean my secondary insurance if I have one?” and “I think that what that is, is if you have two different insurances.”

#### 3.3.2 Health insurance plan decision‐making: Trade‐offs

Survivors considered trade‐offs when making health insurance plan decisions, including those related to OOP costs and specialist referrals. Some survivors were willing to trade off costs for a specialist referral requirement, stating “Even though I need a referral, it still looks like it was a cheaper plan…So yeah it just came down to the dollar.” Cancer severity may also influence this choice, with one survivor stating “I'm looking at the financial cost of it. I think if I was in a worse condition, maybe I'd choose one that had more flexibility and the physicians and specialists that I could select.” However, other survivors disliked the extra steps required for specialist referrals stating, “I go for the least amount of resistance. Meaning, the primary care physician referral requirements if there's none, it's like, ‘Boom. Perfect.’ That's one less obstacle to go through.” Potential care delays due to referrals were also a prominent concern, with one survivor stating, “I prefer not to have a referral. Especially when you're dealing with something like cancer, you want to move fairly quickly. Sometimes that's challenging [with a referral], you know?”

#### 3.3.3 Issues post‐health insurance plan choice: Usability

Overall, survivors felt alone in using their health insurance, saying “You're on your own. You have to do your own investigation. You have to figure it out. You've got to call,” and “Nobody guided me through it. I just did the math and the research.” Survivors also reported confusion regarding OOP costs, stating “I had a really hard time finding out what was going to be covered. I had many long calls where I was on hold for hours at a time with the insurance trying to confirm that the procedures I needed and the tests I needed would be covered…Some things my doctor's office and my surgeon checked coverage on for me, so I wouldn't have to worry about it. And then, I ended up getting a bill for it later anyway. So yeah, they made a stressful, scary experience for me 100x worse,” and “My doctor would say, ‘Okay, we're going to do X, Y, Z.’ And I was like, ‘All right, but is that covered?’ Some of it would only be 80% covered. I just felt it was really confusing and I was kind of just always just not quite sure what was going on.”

## 4 DISCUSSION

This mixed methods study of cancer survivors found that benefit considerations in health insurance decisions are varied and confusing for those with both high and low health insurance literacy. Survivors spent the most time comparing benefit costs related to prescription drugs and ranked OOP maximums and coinsurance as the most important and confusing benefits, respectively. These results reveal an enormous need for health insurance literacy education to prepare survivors to more easily select the optimal plan for themselves and prevent potential cancer‐related financial hardship stemming from insurance plan choices discordant with oncologic care needs. However, few programs exist to educate individuals during health insurance choices. One community‐based health insurance educational program increased health insurance literacy, yet gains in health insurance literacy were inequitable, with individuals who were male, lower income, and living in states that did not show support of the Affordable Care Act (ACA) having smaller or nonsignificant increases in health insurance literacy.^16^ Thus, there is a need for health insurance literacy interventions, such as education delivered by financial navigators,^17^, ^18^ to ensure survivors are able to select the best plan for their needs and ultimately decrease risk of financial hardship.

Cancer survivors in our study with both high and low health insurance literacy ranked the OOP maximum as the most important benefit feature when choosing health insurance plans, knowing they “would have to pay [it]” due to their diagnosis. Our qualitative interviews suggest this importance may be rooted in its ease of calculation, since it was often cited as a benefit used for plan decision‐making. The OOP maximum is a set amount which does not change based on the amount of money spent on healthcare in a plan year. OOP maximums are, in theory, protective for privately insured individuals with cancer. Patients often reach their OOP maximum within the first 1–2 months after diagnosis and are no longer responsible for covered, in‐network costs of care during that benefit year.^19^ However, reaching the annual OOP maximum is often unaffordable.^20^, ^21^ In 2022, the ACA‐mandated limitation for employer‐sponsored or private insurance OOP maximums was $8750 for individuals and $17,400 for family coverage. Previous research has shown that these high limits act as a barrier to cancer care, which may be exacerbated among survivors who are Black or African American, experiencing low income, or enrolled in HDHPs.^22^, ^23^, ^24^, ^25^, ^26^, ^27^ Furthermore, patients could face these high costs annually, since the OOP maximum is a yearly benefit. Though an important benefit in insurance plan decision‐making due to their straightforward calculation of potential costs, policies limiting OOP maximums should be aware of their real‐world affordability in the patients they are designed to protect.

Survivors with both high and low health insurance literacy spent the most time comparing benefit costs related to prescription drugs in both insurance choice sets, with survivors with low health insurance literacy spending a higher proportion of time comparing these costs. This indicates a persistent concern about drug costs. Cost calculations related to prescription drugs are complex and require an understanding of OOP costs associated with different tiers, generic and branded drugs, formulary and non‐formulary drugs, and specialty medications. Even physicians, who are extremely familiar with the healthcare system, have trouble estimating drug costs, with a study of 371 physicians finding only 21% could answer four questions estimating prescription OOP costs correctly.^28^ Difficulty calculating drug costs could lead to unexpected and unmanageable bills for prescription medications, as median inflation‐adjusted prescription drug OOP costs for commercially insured patients have increased by 53% from 2010 to 2016.^29^ Recent legislation from the Inflation Reduction Act has sought to protect patients from high drug costs by capping annual Medicare Part D OOP spending at $2000 starting in 2025, thus saving Medicare beneficiaries an estimated 40% of their annual OOP costs on average.^30^ Though this cap is beneficial for patients, more policy‐oriented work is needed to ensure patients can both afford and easily calculate potential prescription drug costs based on individual insurance policies and annual spending, since high drug prices can lead to cost‐related medication nonadherence and other adverse patient outcomes.

Our quantitative study results revealed that survivors with both high and low health insurance literacy found coinsurance as the most confusing plan benefit, and our qualitative interview findings confirmed survivor confusion surrounding this benefit, stating it is “kind of ambiguous.” Coinsurance is a cost‐sharing mechanism designed to limit unnecessary healthcare utilization by forcing beneficiaries to pay for a portion of the services they use. However, this mechanism largely rests on two assumptions, namely (1) an understanding of the price of healthcare services in order to calculate potential OOP costs, and (2) the ability to decrease or decline healthcare services without health‐related consequences. For cancer survivors, these assumptions are difficult, if not impossible, to meet. Beginning in 2021, Centers for Medicare & Medicaid Services required hospitals to post payer‐negotiated prices online to increase price transparency for patients. However, recent studies have shown that only one in five National Cancer Institute–designated cancer centers abide by this requirement, with wide price variation between centers.^31^, ^32^ It is unclear whether these posted prices are utilized by many patients or aid in better understanding true OOP costs and affordability of care.^33^ Furthermore, individuals with cancer may not respond to transparent prices with reduced utilization, whether high‐ or low‐value care. Therefore, cost sharing may be an inefficient mechanism to incentivize patients to reduce utilization and spending. Instead, efforts to incentivize physicians to select guideline‐appropriate, high‐value care may be more useful to drive down overall healthcare costs.

Results from our study should be considered within the context of several limitations. Survivors included in our sample were highly educated and had already received cancer treatment, and thus may be more aware of cancer‐related costs driving health insurance plan decisions. Survivors were also recruited by a research recruiting firm and may be more engaged in their healthcare. Thus, our results may overestimate the prevalence of high health insurance literacy in the general population of cancer survivors. The health insurance plan choice sets were simulated; however, they were based on actual plans. Survivors also completed the choice sets alone, which may not represent real‐world decision‐making with a family member. Furthermore, eye‐tracking methods are imperfect since data on why survivors dwelt on an area of interest are not immediately captured. However, we attempted to understand survivor dwell time by capturing data on the most important and confusing health insurance plan benefit and through qualitative interviews.

## 5 CONCLUSION

This study of commercially insured cancer survivors found varied benefit considerations in health insurance plan decision‐making for those with both high and low health insurance literacy. OOP maximums and costs surrounding prescription drugs were important in decision‐making, and coinsurance was confusing. Interventions to aid in health insurance understanding, choice, and affordability are needed to optimize plan choice and potentially reduce cancer‐related financial hardship.

## DISCLAIMERS

The opinions expressed by the authors are their own and this material should not be interpreted as representing the official viewpoint of the U.S. Department of Health and Human Services, the National Institutes of Health, or the National Cancer Institute.

## AUTHOR CONTRIBUTIONS

**Courtney P. Williams:** Conceptualization (lead); data curation (lead); formal analysis (lead); funding acquisition (lead); investigation (lead); methodology (lead); project administration (lead); writing – original draft (lead); writing – review and editing (lead). **Heather N. Platter:** Data curation (equal); formal analysis (supporting); funding acquisition (supporting); investigation (supporting); methodology (supporting); writing – review and editing (supporting). **Amy J. Davidoff:** Conceptualization (supporting); formal analysis (supporting); methodology (supporting); writing – review and editing (supporting). **Robin C. Vanderpool:** Conceptualization (supporting); writing – review and editing (supporting). **Maria Pisu:** Investigation (supporting); writing – review and editing (supporting). **Janet S. de De Moor:** Conceptualization (supporting); investigation (supporting); supervision (supporting); writing – review and editing (supporting).

## FUNDING INFORMATION

This study was funded through an internal National Cancer Institute Division of Cancer Control and Population Sciences Collaborative Research Award for Fellows in Training (CRAFT).

## CONFLICT OF INTEREST STATEMENT

The authors have no conflict of interest to declare.

## Supporting information


Table S1.

## Data Availability

The data that support the findings of this study are available on request from the corresponding author. The data are not publicly available due to privacy or ethical restrictions.
