# Supplementary figures and images for: “It's just not easy to understand”: A mixed methods study of health insurance literacy and insurance plan decision‐making in cancer survivors
Source: Cancer Med. 2023 May 23;12(14):15424–34. doi: 10.1002/cam4.6133 (PMC10417217; doi:10.1002/cam4.6133)

Supplemental Figure 1. Study inclusion cascade.


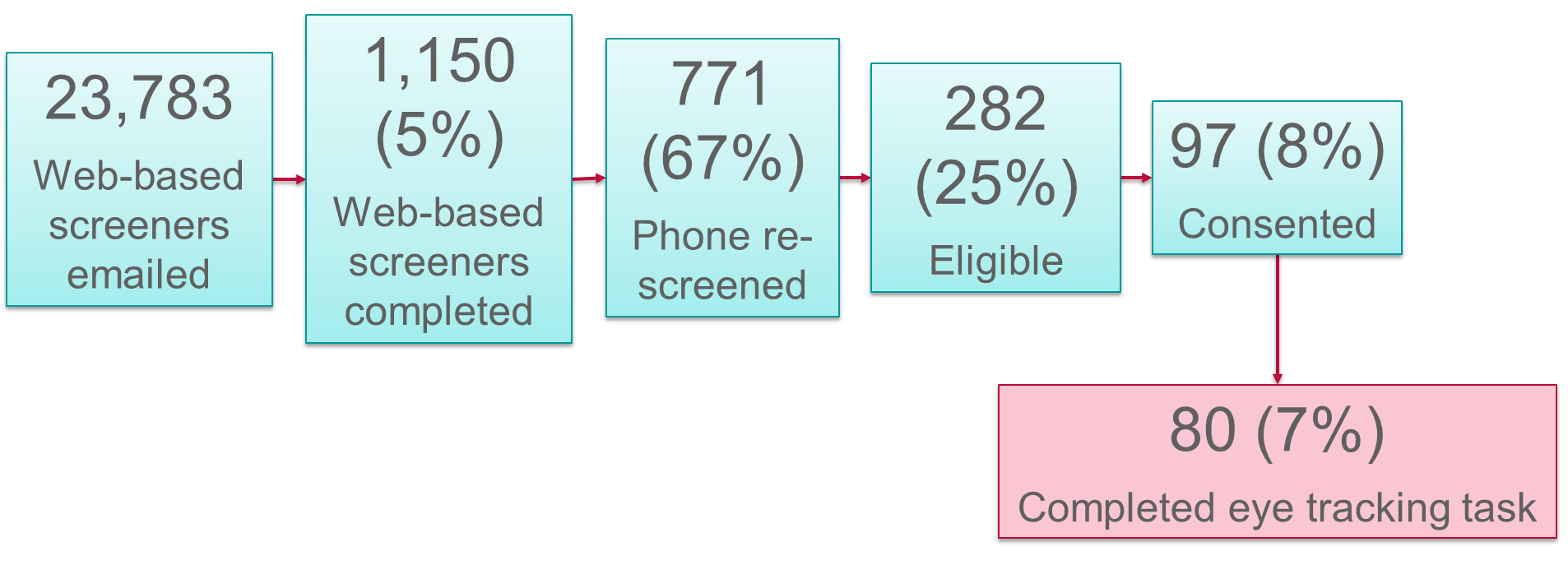

Supplement: Supplementary file 1 — Table S1. [file CAM4-12-15424-s001.docx]
